# Theoretical Calculation and Experimental Verification Demonstrated the Impossibility of Finding Haptens Identifying Triphenylmethane Dyes and Their Leuco Metabolites Simultaneously

**DOI:** 10.3390/molecules23030663

**Published:** 2018-03-15

**Authors:** De-Xin Kong, Fang Lv, Ben Hu, Li-Min Cao

**Affiliations:** 1 State Key Laboratory of Agricultural Microbiology, Huazhong Agricultural University, Wuhan 430070, China; benhu917@gmail.com; 2 Agricultural Bioinformatics Key Laboratory of Hubei Province, College of Informatics, Huazhong Agricultural University, Wuhan 430070, China; 3 Food Safety Laboratory, Ocean University of China, Qingdao 266003, China; lvfang_201712@sina.com; 4 Laboratory of Quality and Safety Risk Assessment for Aquatic Product, Ministry of Agriculture, Aquatic Product Technology Promotion of Beijing, Beijing 100021, China

**Keywords:** triphenylmethane dyes, ELISA, malachite green, crystal violet, molecular modeling

## Abstract

Detection of triphenylmethane dyes (TDs), especially the widely used malachite green (MG) and crystal violet (CV), plays an important role in safety control of aquatic products. There are two chromatic forms of TDs: oxidized or reduced. Usually, only one form can be detected by reported ELISA antibodies. In this article, molecular shape superimposing and quantum mechanics calculation were employed to elucidate the differences between MG, CV, and their reduced chromatic forms (leucomalachite green, LMG and leucocrystal violet, LCV). A potential hapten was rationally designed and synthesized. Polyclonal antibodies were raised through immunizing New Zealand white rabbits and BALB/C mice. We tested the cross-reactivity ratios between the hapten and TDs. The cross-reactivity ratios were correlated with the difference in surface electrostatic potential. The determination coefficients (*r*^2^) of the correlations are 0.901 and 0.813 for the rabbit and mouse antibody, respectively. According to this linear model, the significant difference in the atomic charge seemed to make it impossible to find a hapten that can produce antibodies with good cross-reactivities with both reduced and oxidized TDs.

## 1. Introduction

**Figure 1:**
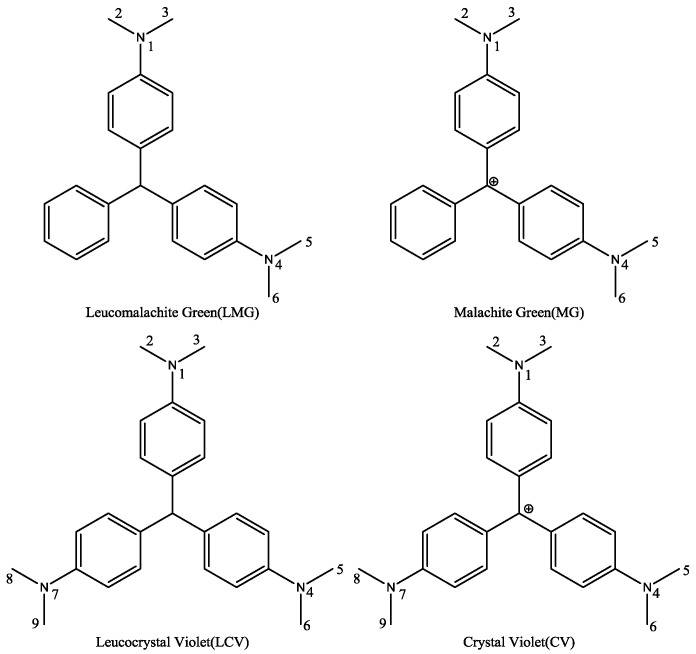
Structures of some representative triphenylmethane dyes.

Triphenylmethane dyes (TDs, Figure 1), including malachite green (MG), crystal violet (CV), and brilliant green (BG), were once widely used in aquaculture for their excellent efficiency to resist bacteria, fungi, and parasites [1,2]. Studies showed that TD residues and their reduced leuco metabolites (leucomalachite green, LMG and leucocrystal violet, LCV) could exist in fish muscles for a long period. These chemicals are highly toxic, carcinogenic, teratogenic, and mutagenic to human beings. Therefore, their application in aquaculture is now strictly prohibited in many countries and regions, including China, the European Union, and the United States [3,4]. Detection of TD compounds, especially the widely used MG and CV, plays an important role in the safety control of aquatic products [5].

Chromatography-based techniques, such as high-performance liquid chromatography (HPLC) and mass spectrometry (MS), are specific, precise, and sensitive and thus are widely employed for the confirmatory analysis of TDs [5,6,7,8]. However, for routine screening of large numbers of samples, enzyme-linked immunosorbent assay (ELISA) and other immunoassays are considered more suitable tools. Besides the significant advantages of rapidness, simplicity, and convenience [1,9], one attractive character of immunoassays is the potential to detect several target molecules simultaneously. The cross-reactivity of antibodies to compounds with a similar structure greatly improved the screening efficiency and reduced the analysis costs. Considering that the monitoring of TD residues is usually based on the total amount of MG, CV, and their metabolites, several studies tried to develop antibodies that can recognize MG, CV, LMG, and LCV with one ELISA kit. However, the produced antibodies usually can only detect the TDs in the same chromatic form as the adopted haptens (Table 1) [1,9,10,11,12,13,14,15,16]. Although the detailed reasons are still unknown, the failure could be largely attributed to the limited understanding of the structure–activity relationship between TD haptens and corresponding antibodies.

The antigen–antibody reaction is a typical molecular recognition process that is dominated by the spatial shape and surface properties of the two counterparts. Therefore, if the shape and surface properties of hapten are similar enough to the compounds under scrutiny, broad reactivity between the antibodies and these compounds may be achieved. There has been increasing attention to the design and improvement of haptens by molecular modeling in the past decade. Such techniques can help one to accurately simulate the structure and predict the physicochemical properties of chemicals [17], and therefore serve as predictive guidance before the experiment or during the interpretation of results afterward [18,19,20]. The theory of molecular modeling can be divided into two branches, quantum mechanics (QM) and molecular mechanics (MM, also called empirical molecular force field). QM is based on electronic structure analysis, and is time-consuming but accurate, while MM uses an empirical force field for the description of atomic interactions, and is fast but not accurate. In 2000 and 2002, Galve and Nichkova calculated the structural similarities among different trichlorophenols and selected the one most similar to the others as the hapten; the success of the strategy was well proved by the broad cross-reactivity of the corresponding antibodies [21,22]. In 2009, with molecular shape superimposing, we studied the family of fluoroquinolones and the results showed a good correlation between the structure similarity of haptens and the cross-reactivity of antibodies [23]. Heretofore, there was no report on the molecular modeling of TDs, nor was there a theoretical study of the relationship between the structure of haptens and the cross-reactivity of antibodies.

In this paper, we studied the hapten’s structure–cross-reactivity relationship by theoretical calculation and experimental verification. We compared the structures of MG, LMG, CV, and LCV with molecular shape analysis. We found that the shapes of the TDs were very similar to each other. The poor cross-reactivity ratio of the previously reported haptens should attribute to the difference in surface electrostatic potential. Then, quantum mechanics calculations were performed to analyze the difference between MG, CV, and their reduced chromatic forms. Haptens were rationally designed as the average surface electrostatic potential of MG, CV, LMG, and LCV. We believe that this can balance the cross-reactivity with the oxidized and reduced TDs. One of the designed haptens was synthesized and conjugated to a carrier protein, and corresponding polyclonal antibodies were raised. The quantitative relationship between the structure of TDs haptens and the cross-reactivity of antibodies was established with a high determination coefficient (*r*^2^ = 0.901 or 0.813). According to this model, the significant difference in the atomic charge seemed to make it impossible to find a hapten that can produce antibodies with good cross-reactivities with both reduced and oxidized TDs. These results should be helpful for analytical chemists in understanding the experimental results of TDs or similar systems.

## 2. Results

### 2.1. Molecular Superposing

There was a significant difference among MG, CV, LMG, and LCV. First, the hybridization form of the central carbon atom was sp^3^ in LMG or LCV and sp^2^ in MG or CV. Therefore, the spatial binding direction of the central carbon atom was different. One of them was a regular tetrahedron, while the other was a planar triangle. Second, for MG and CV, there was a positive charge atom. The formal charge can be assigned to the central carbon atom or amino group nitrogen atom when we draw the 2D structure of the molecules. However, since the molecules are highly conjugated, the charge can distribute to other atoms in the whole molecule. Third, there was a third dimethylamino group attached to the third benzene ring for CV and LCV. Considering that there is no hydroxyl or amino group in TDs, this dimethylamino group is usually modified and used as a link arm to conjugate the protein carriers. Therefore, in principle, the difference of the third amino group will not influence the cross-reactivity ratio of the corresponding antibodies.

**Figure 2:**
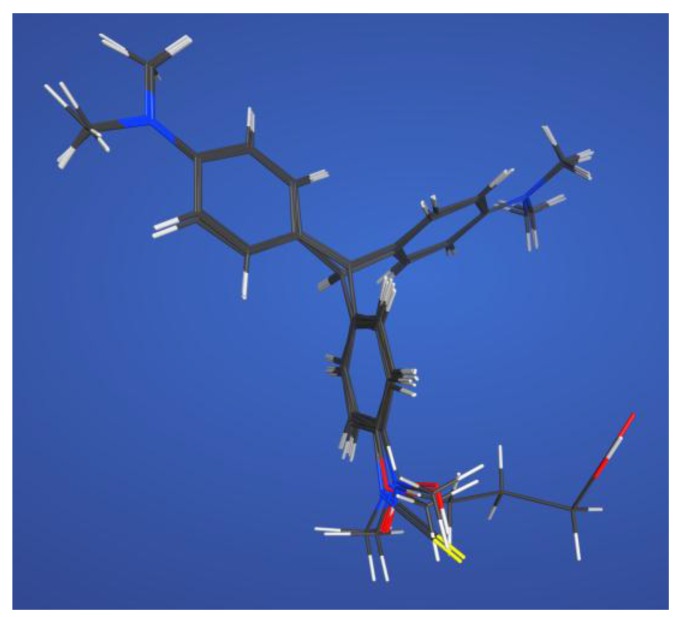
Superimposed structures of MG, CV, LMG, LCV, and the haptens listed in Table 1.

Molecular shape alignment showed that the shapes of the MG analogues were very similar to each other (Figure 2), indicating that the compounds can possess similar steric interactions and fit well spatially in the binding pocket of the antibody. The difference in the hybridization form of the central carbon atom did not cause much molecular shape differentiation. The poor cross-reactivity ratio of the previously reported haptens is mainly due to the difference in surface electrostatic potential.

### 2.2. Ab Initio Calculation and Hapten Design

Ab initio calculation was performed to get the Milliken charge distribution of the TDs and of about 100 MG analogues searched in the databases or rationally designed. Because calculation at different theoretical level gave different results (Supplementary Table S1), a comparison should be performed with the same method and at the same higher-level basis set. In this paper, all results were given based on B3LYP/6-31G* calculation. The carbon atoms in dimethylamine groups were selected as indicative atoms whose charge varied much more than nitrogen atoms. The charge of carbon atoms reflected the surface electrostatic potential.

The calculated partial charge of the indicative atoms in the four TDs is listed in Table 2. The charges of the two dimethylamine groups were almost the same for any of the four TDs. This indicates that the electrostatic structure of TDs is C2 symmetric. The positive charge in MG and CV was distributed into the whole molecule through the conjugation system. For MG and CV, the partial charge of the dimethylamine carbon atoms was 0.229 and 0.217, respectively. For LMG and LCV, the charge of the carbon atoms was 0.153 and 0.151. The atoms’ charge in the reduced form was much lower than that of the oxidized from.

The results of other hapten candidates were consistent with the results mentioned above (Supplementary Table S2). The partial charge of the dimethylamine carbon atoms in hapten **A** or **B** was around 0.23, while the partial charge of carbon atoms in hapten **C** to **F** was around 0.15. These results confirmed our initial hypothesis that the difference in electrostatic potential may be the main reason for the low cross-reactivity between the MG-raised antibody and LMG, and vice versa.

Based on the above analysis, we supposed that compounds with a similar topological structure and with a dimethylamine carbon atom charge at around 0.185 (average value of 0.15 and 0.22) should have a similar cross-reactivity with CV/MG and LMG/LCV. Through substructure searching, over 70 compounds with similar topological structure were found in the ACD, Zinc, and SciFinder databases. More than 30 compounds were designed rationally by adding an electron-repulsive group to MG or an electron-withdrawing group to LMG. The calculated Mulligen charge of reported haptens, some of the TD analogues found in the above databases, and several rationally designed compounds are provided in Supplementary Tables S2–S4. Most of the compounds showed similar charge distribution patterns. The compounds meeting our criteria (their dimethylamine carbon atoms carried charges around 0.185) were marked in green (Supplementary Tables S2–S4). They were selected as hapten candidates. However, most of the candidates lack a hydroxyl or amino group to conjugate a protein carrier to prepare complete antigens, and they are not commercially available. The compound with a very strong electron-withdrawing group (-N+) and atomic charge of 0.1725 was finally selected as the target hapten for further study.

### 2.3. Synthesis of the Hapten and Antigen and Development of the Antibody

The intermediate and designed hapten was successfully synthesized according to 1H-1HCOSY, HMBC, and ESI-MS analysis (Supplementary Figures S1 and S2).

**Figure 3:**
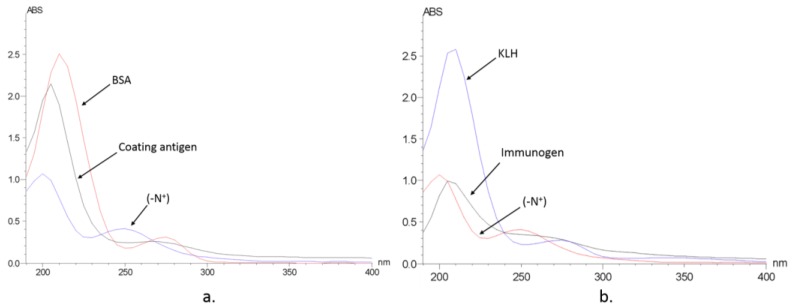
UV spectrum of the designed hapten (-N+), protein and complete antigen. (**a**) BSA as protein; (**b**) KLH as protein.

Immunizing and coating antigen were synthesized through conjugating the hapten to KLH and BSA through the carbodiimide method. The conjugation of BSA and hapten was confirmed with a UV spectrum (Figure 3), gel electrophoresis, and Ultraflex II MALDI-TOF/TOF-MS. UV scanning showed the absorbance peaks of N+ group were around 200 nm and 250 nm. The absorbance peaks of BSA were at 220 nm and 278 nm. The antigen showed an overlaid feature, from which the coupling ratio should be around 7.51. Gel electrophoresis proved the mass of antigen was higher than the carrier protein BSA. According to the results of Ultraflex II MALDI-TOF/TOF-MS, the mass of BSA and the BSA–hapten conjugate was calculated as 65996.2 and 69430.0, respectively, and therefore the coupling ratio should be about 7.23:1 (hapten:BSA). All the results were consistent and confirmed the successful preparation of the coating antigen. The envelope antigens were successfully prepared, since the three methods were consistent. Similar to coating antigens, the effective conjugation between KLH and haptens was confirmed by the UV spectrum, and the coupling ratio (hapten:KLH) was estimated at 14.59:1. Polyclonal antibodies were raised through immunizing New Zealand white rabbits and BALB/C mice, and the titers of antiserum were determined to be 51,200 and 12,800, respectively.

### 2.4. Cross-Identification Efficiency

**Figure 4:**
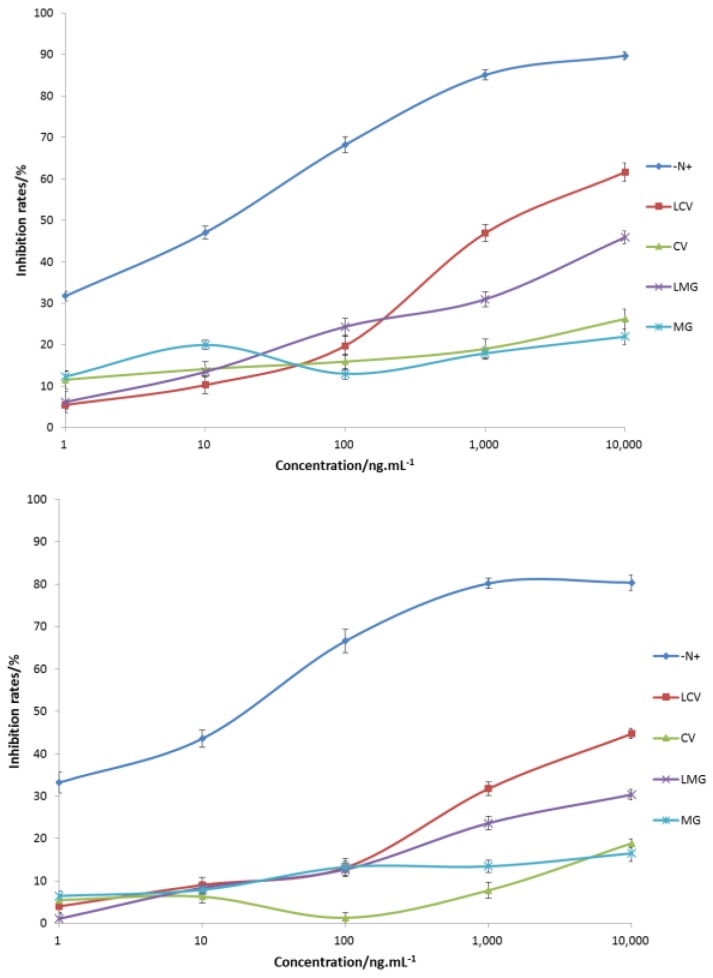
Ci-ELISA curves for rabbit (**top**) and mice antibodies (**bottom**). Repeated three times.

The cross-reactivity of prepared antibodies were determined and summarized (Figure 4). The antibodies could specifically recognize the synthetic antigen (N+) with half inhibition rates in the range of 10–100 ng·mL^−1^. The recognition of MG analogues was weak, and a decreased trend was observed in the order LCV, LMG, CV, and MG (Figure 4). Obviously, the reason for the weak recognition could be the electrostatic difference between the hapten and MG analogues.

**Figure 5:**
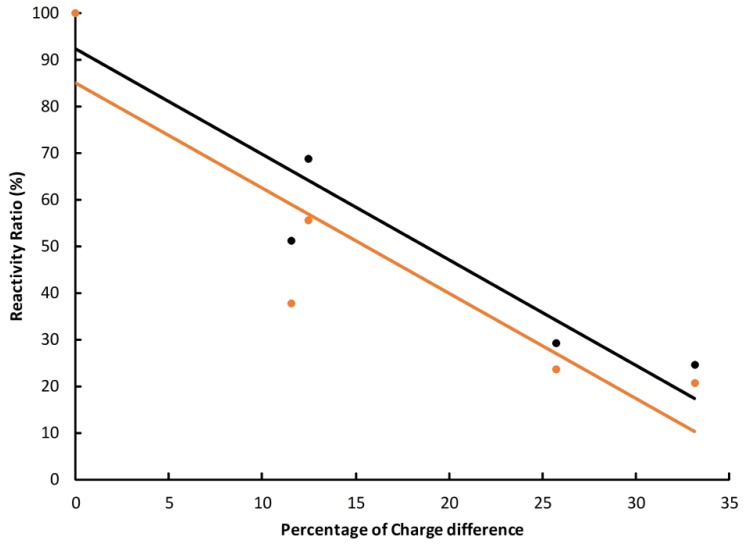
Structure-cross-reactivity relationship of the rabbit (black) and mice (orange) antibodies.

Then the structure-activity relationship of the antigen and antibody was analyzed with four TDs. Their charge difference compared to the hapten was used as the abscissa and the cross-reactivity ratio calculated at a concentration of 10^4^ ng·mL^−1^ was used as the ordinate. The results showed that there was a good linear correlation between the charge difference and the cross-reactivity ratio (Figure 5 and Supplementary Table S5). The determination coefficient (*r*^2^) was 0.901 and 0.813 for rabbit and mouse antibodies, respectively. The overall trend was consistent with the prediction model of charge distribution, which confirmed our initial hypothesis.

## 3. Discussion

In this paper, molecular shape superimposing and quantum mechanics calculations were performed to elucidate the difference between MG, CV, and their leuco metabolites, LMG and LCV. The shapes of MG, CV, LMG, and LCV were found to be similar, but the surface electrostatic potential of MG and CV is much higher than that of LMG and LCV. Based on the above observation, a potential hapten with average atomic charge was designed and synthesized. Just as expected, a high correlation (determination coefficient *r*^2^ over 0.8) between the cross-reactivity ratio and the charge difference (carbon atoms) was observed, which proved our initial hypothesis and confirmed the feasibility of our approach in hapten design.

However, the cross-reactivity ratios of the hapten with TDs were very weak, which means they are not applicable to real residue detecting. Calculated with the above linear model, the cross-reactivity for the ideal hapten (with a carbon atom charge of 0.185) will be around 40%, which is very weak. Considering that polyclonal antibodies represent the average property of numerous cells, we suggest the atomic charge of haptens plays the dominant role in the specificity of corresponding antibodies. This result indicated that the significant difference in the atomic charge seems to make it very difficult or even impossible to find a hapten that can produce polyclonal antibodies recognizing both reduced and oxidized TDs. Considering the possible individual differences among cells, there may be exceptions in certain monoclonal antibodies, though the chance seems very low. The total charge of the hapten is possibly an important factor, since the reduced form of TDs is neutral and oxidized they have a +1 positive charge. Further studies with designed neutral haptens may provide more clues.

The conclusion in this paper is consistent with most of the studies listed in Table 1 [1,14,16,24], in which the antibodies can recognize only one chromatic form of TDs, oxidized or reduced. Therefore, professional preprocessing is still required to convert MG and CV to their reduced form or the other way around. Two references reported antibodies with high cross-reactivity ratio values [9,25], which was not supported by our theoretical analysis and also was opposite to the study results with the same hapten (Table 1) [12,13]. Though detailed proof is still limited, the reasons may be as follows: (i) the genetic difference in the experimental animals for preparation of antibodies; (ii) the conjugation with carrier proteins may cause some post-conjugation changes of haptens, for example by hydrogen bonds, van der Waals forces, hydrophobic interactions, and electronic bonds, which are still difficult to accurately evaluate with the existing techniques but will affect the properties of corresponding antibodies; (iii) there may be accidental sample contamination or chemical changes in the preparation of complete antigens and coating antigens, or immunization of animals—that is to say, more than one form of haptens were introduced into the system and therefore resulted in false cross-reactivity.

This paper shows the great impact of charge distribution on molecular recognition and proved that, besides the molecular shape, surface electrostatic potential is also important for the antigen and antibody binding. This result is meaningful for molecular design, including hapten design, and helpful for analytical chemists in understanding the experimental results of TDs or similar systems.

## 4. Materials and Methods

### 4.1. Molecular Modeling and Superimposing

Three-dimensional structures of MG, LMG, CV, LCV, and their derivatives that were reported as haptens or analogues were built in Molecular Operation Environment (MOE) program (2012.10, Chemical Computing Group, Montreal, QC, Canada) [26]. The structures were optimized with a MMFF94 force field and a high convergence restriction of RMS gradient (0.001 kcal/mol). Then, the molecules were superimposed with atom-based RMS optimization, followed by flexible alignment with MOE’s Flexible Alignment module. The molecule alignment was performed by maximizing steric and feature overlap while minimizing internal ligand strain. During the alignment process, methods including rigid, flexible alignment and refining of existed alignment were used sequentially to obtain a reasonable overlap of the structures.

### 4.2. Ab Initio and Density Functional Calculation

From the above analysis, we found that the TDs’ shapes are similar to each other (Figure 2). Therefore, the main difference between the two forms lies in their electrostatic potential, which subsequently caused weak cross-reactivity. As mentioned in the introduction, the electronic structure can only be precisely calculated with ab initio QM methods.

The inputted molecules were then optimized, step-by-step, with the semi-empirical AM1 method and HF self-consistent field method with basis sets of 3-21G, 6-31G, and 6-31G**. The optimized structures were further optimized with the Becke three-parameter, hybrid functional combined with the Lee, Yang, and Parr correlation functional (B3LYP) method and basis sets at the 6-31G* level. AM1 calculation was performed with MOE and all the ab initio and density functional calculations (B3LYP) were performed with the Gaussian 03 program suite (Revision B.2, Gaussian Inc., Pittsburgh, PA, USA) [27]. Milliken population analysis was performed to calculate the partial charge of the atoms. The charge of hydrogen was assigned to its linked heavy atoms. Four atoms, i.e., the carbon atoms in the amino group (atoms with numbers in Figure 1), were selected as representative to analyze the surface electrostatic potential. The carbon atoms of a potential hapten should carry charges around the average value of the oxidant and reductive forms of CV or MG.

Two strategies were used to find or design possible haptens. First, similarity or substructure searching was performed against the MDL ACD, SciFinder, and Zinc databases [28]. Secondly, electron-donating and electro-withdrawing groups were added to the scaffold of MG and LMG, respectively. The charge distribution of the compounds returned by structure searching and the designed compounds were calculated with the same protocol described above. Finally, about 10 compounds were selected as hapten candidates. However, none of the compounds was commercially available. They also lack a suitable group to link to the serum protein.

### 4.3. Synthesis of the Hapten

**Figure 6:**
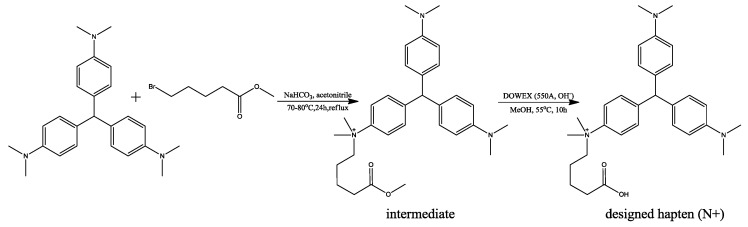
Synthesis protocol of the designed hapten (N+).

Since no potential compounds can be purchased from the suppliers, one designed hapten (marked as N+) was synthesized in two steps (Figure 6), according to the procedures described in the references with some modifications [29,30].

Firstly, the intermediate in Figure 6 was synthesized from LCV through a nucleophilic reaction. Leucocrystal violet (1.8675 g, 5.0 mmol), methyl-5-bromovalerate (5.0 mmol, *d* = 1.363, 0.697 mL), and NaHCO_3_ (20.0 mmol, 1.68 g) were added to anhydrous acetone (50.0 mL). The mixture was heated with refluxing for 24 h at 70–80 °C. Then, the mixture was filtrated immediately and washed with hot acetone. The solution was evaporated under a stream of nitrogen, and purified by silica gel column-layer chromatography with ethanol:triethylamine (10:1) to get the intermediate. The structure of the synthesized intermediate was confirmed with 1H-1HCOSY, HMBC, and ESI-MS (Supplementary Figure S1). ^1^H NMR (DMSO-*d*_6_, 500 MHz):*δ*_H_ 7.81 (2H, *d*, *J* = 7.8 Hz), *δ*_H_ 7.29 (2H, *d*, *J* = 7.8 Hz), *δ*_H_ 6.66 (4H, *d*, *J* = 6.7 Hz), *δ*_H_ 6.90 (4H, *d*, *J* = 6.7 Hz), *δ*_H_ 2.84 (6H, s), *δ*_H_ 3.85 (6H, s), *δ*_H_ 3.88 (2H, m), *δ*_H_ 1.33 (2H, m), *δ*_H_ 1.45 (2H, m), *δ*_H_ 2.29 (2H, m), *δ*_H_ 3.52 (3H, s). ESI-MS, calculated for C_31_H_42_N_3_O_2_^+^: 488.33, found: *m*/*z* 488.1 [M]^+^.

Secondly, the intermediate (77.77 mg, 0.137 mmol) and DOWEX (550A, OH^−^, 327 mg) were dissolved in anhydrous methanol (2.0 mL) and stirred at 55 °C for 24 h. The reaction product was cooled to room temperature and washed with methanol. Finally, the pH of the product was adjusted to lower than 7 by adding HCl (1 M). The solution was evaporated under a stream of nitrogen, and purified by silica gel column-layer chromatography with ethanol:glacial acetic acid (5:1) to give the target product. The structure of the synthesized hapten was confirmed with 1H-1HCOSY, HMBC and ESI-MS (Supplementary Figure S2). ^1^H NMR (MeOH-*d*_4_, 500 MHz):*δ*_H_ 7.88 (2H, *d*, *J* = 7.8 Hz), *δ*_H_ 7.4 (2H, *d*, *J* = 7.8 Hz), *δ*_H_ 7.39 (4H, *d*, *J* = 6.7 Hz), *δ*_H_ (4H, *d*, *J* = 6.7 Hz), *δ*_H_ (6H, s), *δ*_H_ (6H, s), *δ*_H_ 3.99 (2H, m), *δ*_H_ 1.52 (2H, m), *δ*_H_ 1.52 (2H, m), *δ*_H_ 2.30 (2H, m). ESI-MS, calculated for C_30_H_40_N_3_O_2_^+^: 474.31, found: *m*/*z* 474.3 [M]^+^.

### 4.4. Preparation of Immunogen and Coating Antigen

The synthesized hapten was conjugated to carrier protein KLH and BSA by the carbodiimide method, synthesizing immunogen, and coating antigen. First, the carrier protein was covered with EDA (200 µL) and EDC (256 mg) in 10 mL of phosphate buffer (0.01 M, pH = 7.0); the mixture was stirred at room temperature for 12 h and dialyzed against phosphate buffer (pH 7.4) to remove the free reagents. Then, 6 mg hapten (N+), 2.08 mg NHS, and 3.72 mg EDC were dissolved in 0.5 mL of DMF and stirred at room temperature for 1.5 h, followed by standing and layering. Finally, the supernatant was added dropwise to the above solution, including 10 mg of the carrier protein. The reaction was incubated overnight at 4 °C under gentle stirring. The solution was centrifuged for 30 s at a speed of 3000 rpm and the supernatant was dialyzed in phosphate buffer (pH = 7.4) for 2 days with the gradient concentrations of DMF until completion. The conjugated immunogen and the coating antigen were verified with UV scanning (Figure 3). The conjugation condition will be judged by calculating the coupling ratio, which is equal to the ratio of the molar concentration of the antigen to the molar concentration of the protein. The coating antigen was further verified with sodium dodecyl sulfate polyacrylamide gel electrophoresis (SDS-PAGE) (BIO-RAD, Hercules, CA, USA) and Ultraflex II MALDI-TOF/TOF-MS (Bruker Daltonics Inc., Madison, WI, USA), which confirmed the validation of UV scanning (Supplementary Figure S3). The standard curves of the hapten, BSA, and KLH are provided in Supplementary Figure S4.

### 4.5. Production of Polyclonal Antibodies

Polyclonal antibodies were raised through immunizing New Zealand white rabbit and BALB/C mouse with immune antigen. The first immunization was treated with immunogen containing a dose of 200 μg, which was dissolved in 200 μL of physiological saline and emulsified with the same volume of Freund’s complete adjuvant. Three subsequent immunizations were given at intervals of 14 days, changing the complete adjuvant into an incomplete adjuvant. The anti-serum was harvested a week after the last immunization and stored at −20 °C until required. The titers of anti-serum were monitored by indirect ELISA. For detailed information about this step, please refer to [9,13].

The optimal working concentration was optimized with a checkerboard study. The concentration of envelope antigen was 1 µg∙mL^−1^, the dilution ratios of goat anti-rabbit IgG secondary antibody and goat anti-mouse IgG secondary antibody was 5000 and 10,000 respectively (Supplementary Table S6).

### 4.6. Cross-Identification Efficiency

The cross-reactivity experiment was used to evaluate the specificity of antibodies by indirect competitive ELISA [9,13]. The synthesized antigen was diluted with phosphate buffer at the required concentrations of 1, 10, 10^2^, 10^3^, and 10^4^ ng∙mL^−1^. Malachite green, crystal violet, and their metabolites were prepared at the same concentrations. The testing was carried out with a blank control and negative control, and repeated three times. The cross-reactivity (CR) is usually calculated based on the inhibition rate of 50% (IC_50_), but in this paper the inhibition rate of all analytes was less than 50%. Therefore the inhibition rate at the highest test concentration (10^4^ ng·mL^−1^) was used and the CR was calculated with the following formula:

CR (%) = [(inhibition rate of antigen)/(inhibition rate of the given analyte)] × 100%.

Then, the structure–cross-reactivity relationship of the antigen and antibody was analyzed by comparing the inhibition rate under concentration of 10^4^ ng·mL^−1^ and the charge difference of the four ingredients compared with the designed hapten.

## Figures and Tables

**Table 1:**
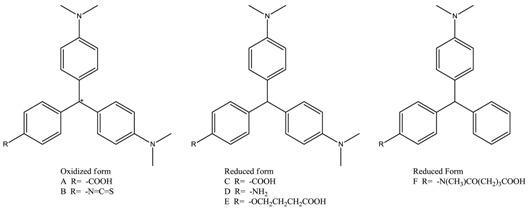
Structures and cross-reactivity ratios of reported haptens with MG, LMG, CV, and LCV.

Hapten	MG	LMG	CV	LCV	Reference
A	100%	<0.01%	100%	<0.01%	[1]
A	100%	-	95%	-	[14]
A	100%	<0.1%	98%	<0.1%	[16]
B	100%	1%	42%	<0.01%	[11]
C	~3%	100%	<0.01%	200%	[1]
D	95.25%	100%	29.07%	212.38%	[9]
D	24.33%	100%	-	-	[13]
D	13.5%	100%	5.89%	40.67%	[12]
E	26.43%	100%	-	-	[10]
F	12%	100%	0.8%	2.4%	[15]

**Table 2:** Partial charges of the indicative atoms in MG, LMG, CV, LCV, and the designed hapten calculated at the B3LYP/6-31G* level.

Atom *	MG	LMG	CV	LCV	N+
1 N	−0.464	−0.472	−0.467	−0.473	−0.474
2 C	0.231	0.152	0.217	0.152	0.176
3 C	0.229	0.153	0.217	0.152	0.170
4 N	−0.464	−0.472	−0.467	−0.472	−0.474
5 C	0.231	0.153	0.217	0.150	0.171
6 C	0.229	0.152	0.217	0.150	0.173
7 N			−0.467	−0.473	
8 C			0.217	0.152	
9 C			0.217	0.152	
Average charge of C atoms	0.230	0.153	0.217	0.151	0.173

* Atomic numbers were marked in Figure 1.

## Supplementary Materials

Supplementary materials are available online.

## References

[1] Yang M.C., Fang J.M., Kuo T.F., Wang D.M., Huang Y.L., Liu L.Y., Chen P.H., Chang T.H. (2007). Production of antibodies for selective detection of malachite green and the related triphenyl methane dyes in fish and fishpond water. J. Agric. Food Chem..

[2] Bilandzic N., Varenina I., Kolanovic B.S., Oraic D., Zrncic S. (2012). Malachite green residues in farmed fish in Croatia. Food Control.

[3] Hurtaud-Pessel D., Couedor P., Verdon E., Dowell D. (2013). Determination of residues of three triphenylmethane dyes and their metabolites (malachite green, leuco malachite green, crystal violet, leuco crystal violet, and brilliant green) in aquaculture products by LC/MS/MS: First action 2012.25. J. AOAC Int..

[4] Conti G.O., Copat C., Wang Z.H., D’Agati P., Cristaldi A., Ferrante M. (2015). Determination of illegal antimicrobials in aquaculture feed and fish: An ELISA study. Food Control.

[5] Bergwerff A.A., Scherpenisse P. (2003). Determination of residues of malachite green in aquatic animals. J. Chromatogr. B.

[6] Arroyo D., Ortiz M.C., Sarabia L.A., Palacios F. (2009). Determination and identification, according to European Union Decision 2002/657/EC, of malachite green and its metabolite in fish by liquid chromatography-tandem mass spectrometry using an optimized extraction procedure and three-way calibration. J. Chromatogr. A.

[7] Valle L., Diaz C., Zanocco A.L., Richter P. (2005). Determination of the sum of malachite green and leucomalachite green in salmon muscle by liquid chromatography-atmospheric pressure chemical ionisation-mass spectrometry. J. Chromatogr. A.

[8] Dowling G., Mulder P.P.J., Duffy C., Regan L., Smyth M.R. (2007). Confirmatory analysis of malachite green, leucomalachite green, crystal violet and leucocrystal violet in salmon by liquid chromatography-tandem mass spectrometry. Anal. Chim. Acta.

[9] Xing W.W., He L., Yang H., Sun C.J., Li D.W., Yang X.M., Li Y., Deng A.P. (2009). Development of a sensitive and group-specific polyclonal antibody-based enzyme-linked immunosorbent assay (ELISA) for detection of malachite green and leucomalachite green in water and fish samples. J. Sci. Food Agric..

[10] Wang J., Han Y., Shen B. (2007). Malachite Green Derivatives for Immunoassay Reagents to Detect Malachite Green. U.S. Patent.

[11] Benchikh E., McConnell R., Fitzgerald S., Lowry A. (2007). Immunoassay Method and Kit To Leucomalachite Green and Malachite Green. U.S. Patent.

[12] Wang Q., Guo D.H., Li J., Jiang W., Zhao X.Y. (2010). Establishment of an indirect competitive enzyme-linked immunosorbent assay for detecting the residues of malachite green. Anim. Husb. Vet. Med..

[13] Zhao C.C., Liu Y.J., Xu B.X., Yang T.T., Wu J., Shen W.Y., Zhang L.S., Sun X.L., Zhao X.L. (2009). Preparation and identification of anti-leucomalachite green antibodies. Chin. J. Health Lab. Technol..

[14] Oplatowska M., Connolly L., Stevenson P., Stead S., Elliott C.T. (2011). Development and validation of a fast monoclonal based disequilibrium enzyme-linked immunosorbent assay for the detection of triphenylmethane dyes and their metabolites in fish. Anal. Chim. Acta.

[15] Singh G., Koerner T., Gelinas J.M., Abbott M., Brady B., Huet A.C., Charlier C., Delahaut P., Godefroy S.B. (2011). Design and characterization of a direct ELISA for the detection and quantification of leucomalachite green. Food Addit. Contam..

[16] Xu H.Y., Chen X.L., Guo L., Zhang J.W., Lai W.H., Aguilar Z.P., Wei H., Xiong Y.H. (2013). Monoclonal antibody-based enzyme-linked immunosorbent assay for detection of total malachite green and crystal violet residues in fishery products. Int. J. Environ. Anal. Chem..

[17] Broughton H.B. (1997). Molecular modeling. Curr. Opin. Chem. Biol..

[18] Liu Y.H., Jin M.J., Gui W.J., Cheng J.L., Guo Y.R., Zhu G.N. (2007). Hapten design and indirect competitive immunoassay for parathion determination: Correlation with molecular modeling and principal component analysis. Anal. Chim. Acta.

[19] Mu H.T., Lei H.T., Wang B.L., Xu Z.L., Zhang C.J., Ling L., Tian Y.X., Hu J.S., Sun Y.M. (2014). Molecular modeling application on hapten epitope prediction: An enantioselective immunoassay for ofloxacin optical isomers. J. Agric. Food Chem..

[20] Von Kreudenstein T.S., Lario P.I., Dixit S.B. (2014). Protein engineering and the use of molecular modeling and simulation: The case of heterodimeric fc engineering. Methods.

[21] Galve R., Camps F., Sanchez-Baeza F., Marco M.-P. (2000). Development of an immunochemical technique for the analysis of trichlorophenols using theoretical models. Anal. Chem..

[22] Nichkova M., Galve R., Marco M.P. (2002). Biological monitoring of 2,4,5-trichlorophenol (i): Preparation of antibodies and development of an immunoassay using theoretical models. Chem. Res. Toxicol..

[23] Cao L.M., Kong D.X., Sui J.X., Jiang T., Li Z.Y., Ma L., Lin H. (2009). Broad-specific antibodies for a generic immunoassay of quinolone: Development of a molecular model for selection of haptens based on molecular field-overlapping. Anal. Chem..

[24] Shen Y.D., Deng X.F., Xu Z.L., Wang Y., Lei H.T., Wang H., Yang J.Y., Xiao Z.L., Sun Y.M. (2011). Simultaneous determination of malachite green, brilliant green and crystal violet in grass carp tissues by a broad-specificity indirect competitive enzyme-linked immunosorbent assay. Anal. Chim. Acta.

[25] Jiang Y.S., Chen L., Hu K., Yu W.J., Yang X.L., Lu L.Q. (2015). Development of a fast ELISA for the specific detection of both leucomalachite green and malachite green. J. Ocean Univ. China.

[26] Chemical Computing Group (2012). Molecular Operating Environment (MOE), Version 2012.10.

[27] Gaussian Inc. (2003). Gaussian, Version 03.B02.

[28] Irwin J.J., Shoichet B.K. (2005). Zinc—A free database of commercially available compounds for virtual screening. J. Chem. Inf. Model..

[29] Snider B.B., Gu Y.H. (2001). Total synthesis of (−)- and (+)-dysibetaine. Org. Lett..

[30] Albrecht M., Müller M., Mergel O., Rissanen K., Valkonen A. (2010). Ch-directed anion–π interactions in the crystals of pentafluorobenzyl-substituted ammonium and pyridinium salts. Chem. Eur. J..

