# Supplementary material for: Theoretical Calculation and Experimental Verification Demonstrated the Impossibility of Finding Haptens Identifying Triphenylmethane Dyes and Their Leuco Metabolites Simultaneously
Source: Molecules. 2018 Mar 15;23(3):663. doi: 10.3390/molecules23030663 (PMC6017731; doi:10.3390/molecules23030663)
Supplement: Supplementary file 1 [file molecules-23-00663-s001.pdf]

Supplementary Materials

Theoretical calculation and experimental verification demonstrated the impossibility of finding haptens to identify triphenylmethane dyes and their leuco metabolites simultaneously

De-Xin Kong[a, b, #, *], Fang Lv[c, d, #] Ben Hu[a, b] and Li-Min Cao[c,*]

[a] State Key Laboratory of Agricultural Microbiology, Huazhong Agricultural University, Wuhan 430070, China; benhu917@gmail.com (B.H.)
[b] Agricultural Bioinformatics Key Laboratory of Hubei Province, College of Informatics, Huazhong Agricultural University, Wuhan 430070, China
[c] Food Safety Laboratory, Ocean University of China, Qingdao 266003, China; lvfang_201712@sina.com(F.L.)
[d] Laboratory of Quality&Safety Risk Assessment for Aquatic Product, Ministry of Agriculture, Aquatic Product Technology Promotion of Beijing, Beijing 100021, China
[#] The first two authors contributed equally
* Correspondence: dxkong@mail.hzau.edu.cn (D.X.K); caolimin@ouc.edu.cn (L.M.C);
Tel.: +86-27-8728-0877 (D.X.K); +86-532-8203-2389(L.M.C)

Supplementary Information Figures 2
Figure S1 Structure validation of the synthesized intermediate.......... 2
Figure S2 Structure validation of the synthesized hapten (N+). 3
Figure S3 Validation of the coating antigen. 4
Figure S4 The standard curve of antigen with -N+ structure and proteins (BSA and KLH) 5
Supplementary Information Tables 6
Table S1 Calculated partial charge of MG's dimethylamino atoms at different theoretical levels 6
Table S2 Calculated partial charge of dimethylamino carbon atoms of five reported haptens 7
Table S3 Calculated partial charge of dimethylamino carbon atoms of TDs analogues from SciFinder, ACD or Zinc database 8
Table S4 Calculated partial charges of haptens' carbon atoms for the designed haptens 12
Table S5 Structure-cross reactivity relationship of the hapten with TDs 17
Table S6 Working dilution of coating antigen and goat IgG secondary antibody by checkerboard test (n=3) 18

Supplementary Figures

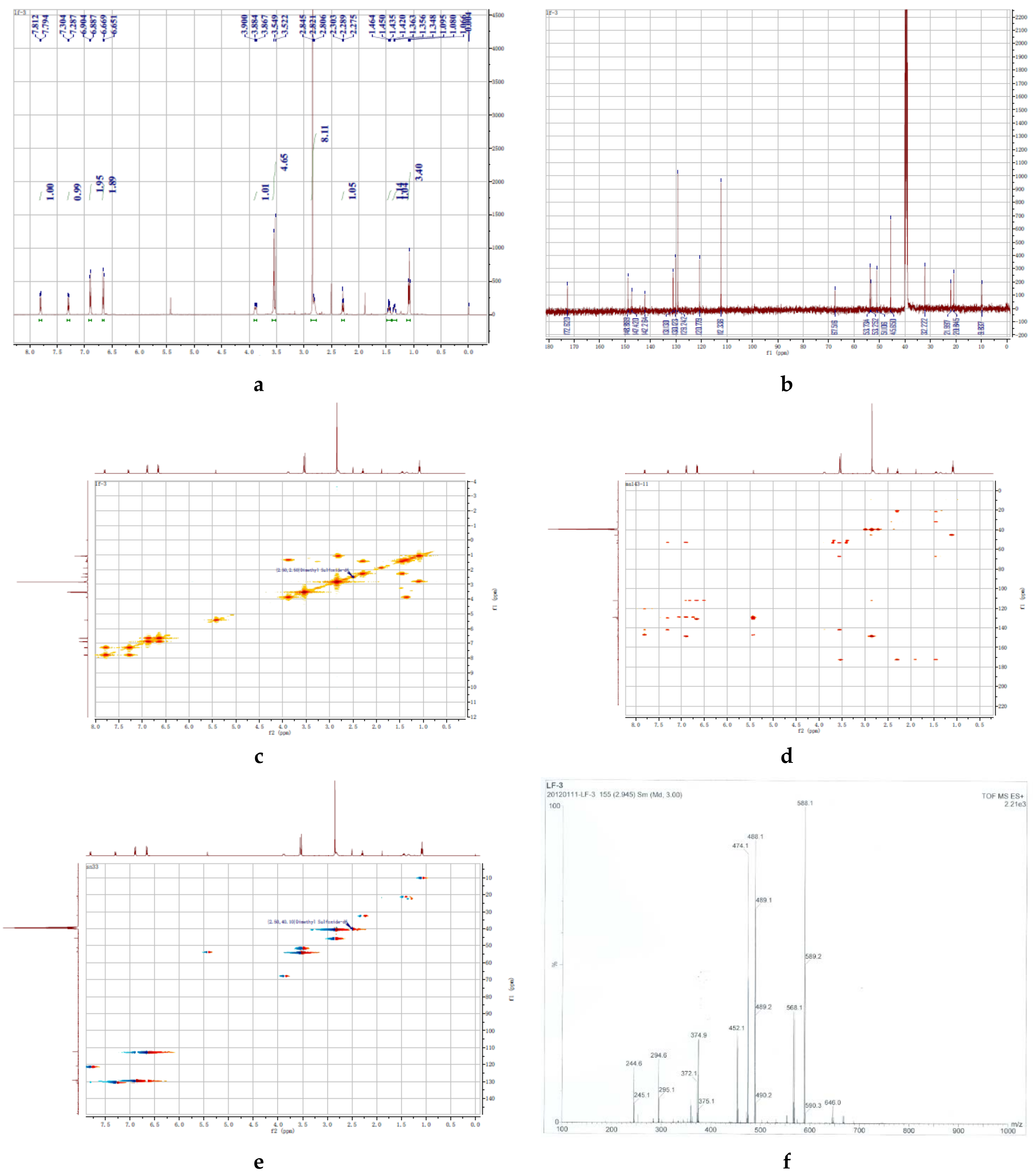

Figure S1. Structure validation of the synthesized intermediate.

a. 1H NMR. b. 13C NMR. c. 1H-1H COSY. d. HMBC. e. HSQC. f. TOF- mass spectra.

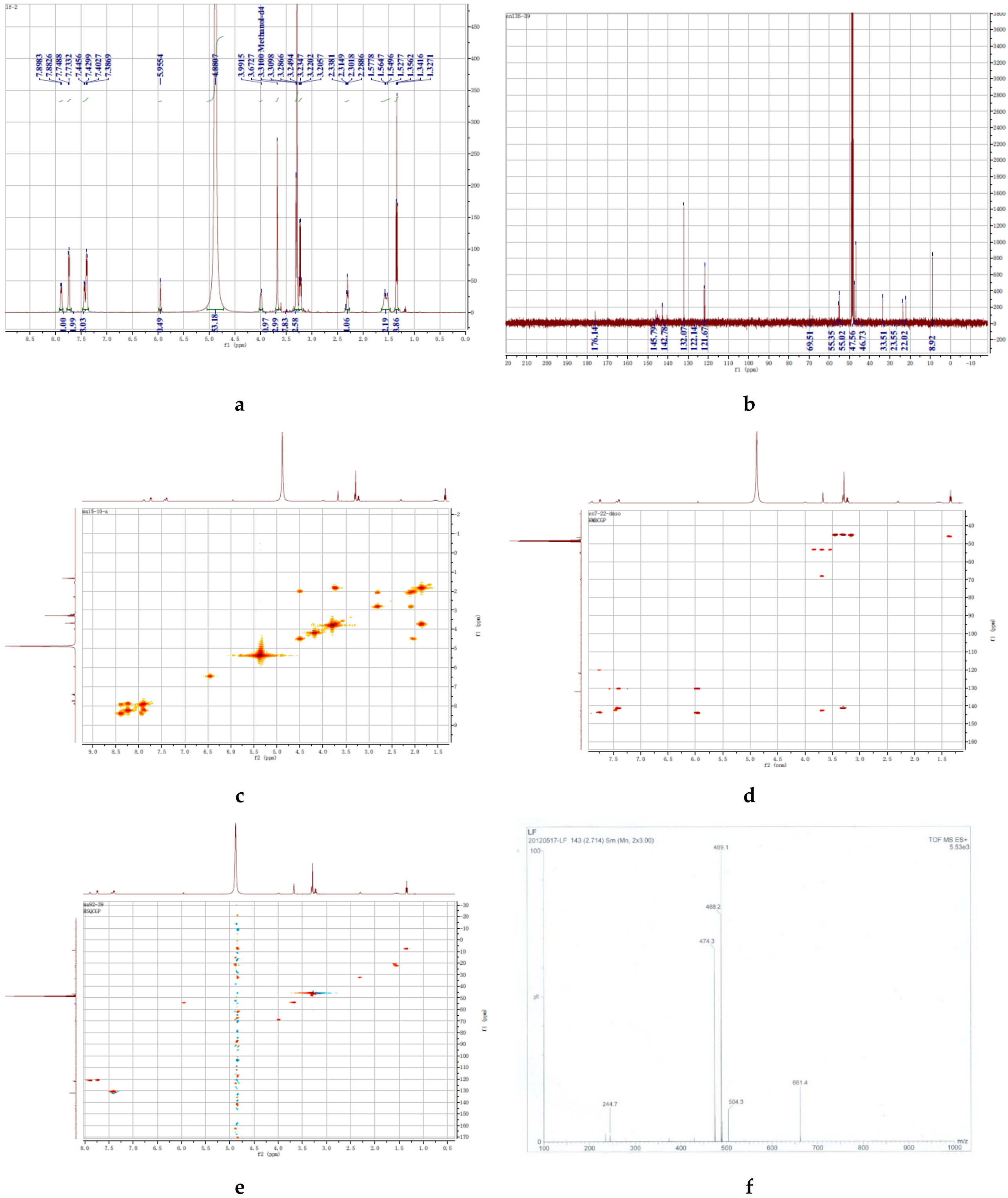

Figure S2. Structure validation of the synthesized hapten (N+).

a. 1H NMR. b. 13C NMR. c. 1H-1H COSY. d. HMBC. e. HSQC. f. TOF- mass spectra.

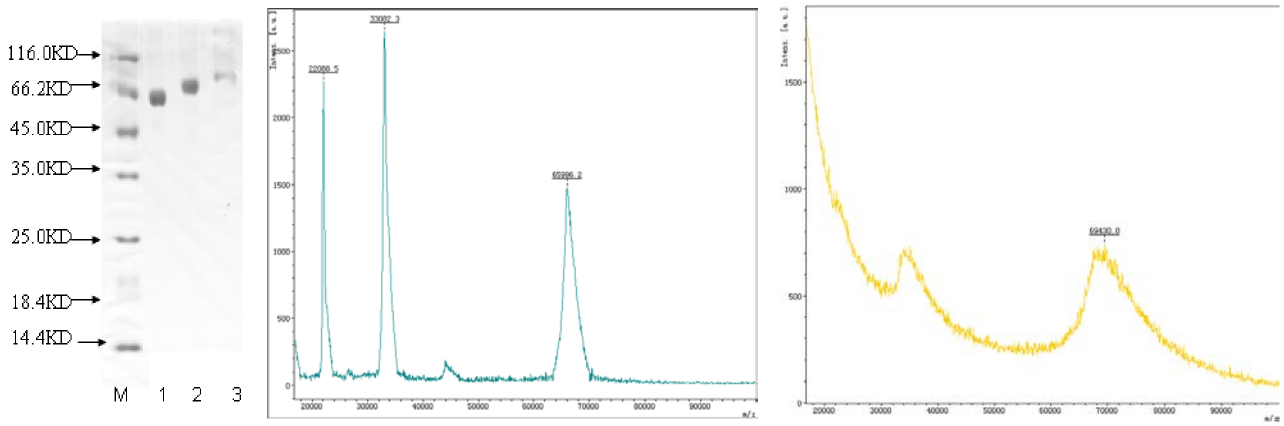

a b c

Figure S3. Validation of the coating antigen.

a. The SDS-PAGE of BSA, cBSA and complete antigen. Marker:low molecular standard protein; 1:BSA; 2:cBSA; 3: complete antigen. b. Ultraflex II MALDI-TOF/TOF-MS of BSA. C. Ultraflex MALDI-TOF/TOF-MS of coating antigen.

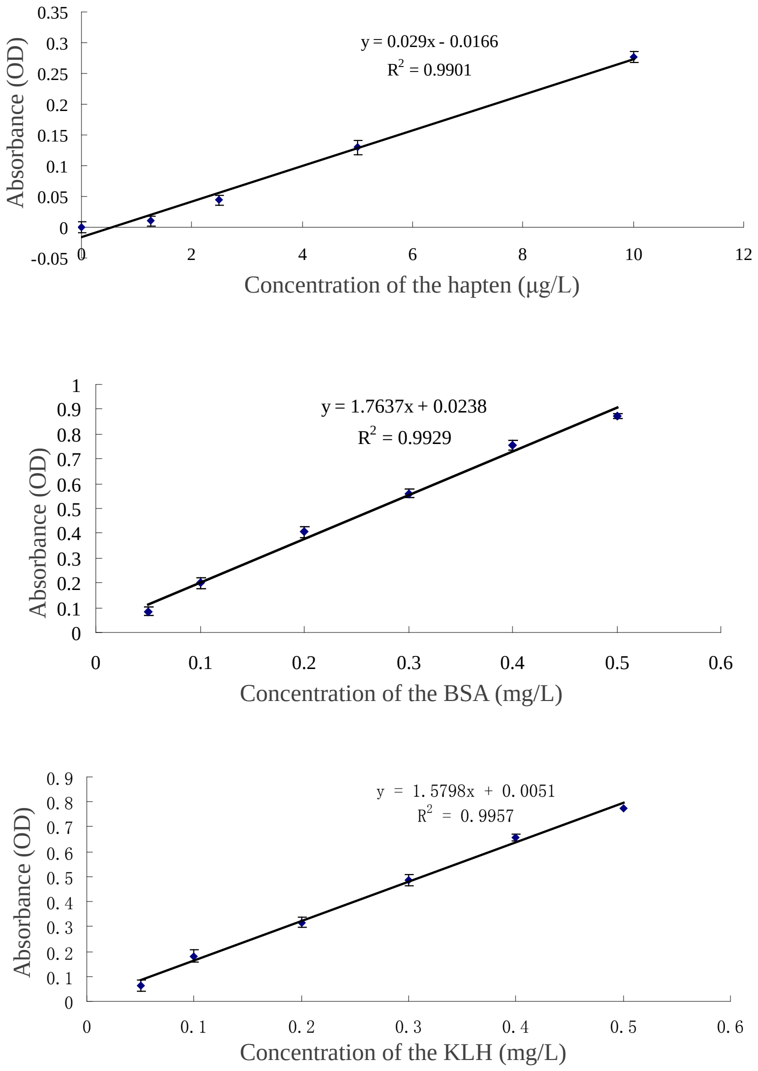

Figure S4. The standard curve of antigen with -N+ structure and proteins (BSA and KLH).

Supplementary Tables

Table S1. Calculated partial charge of MG's dimethylamino atoms at different theoretical levels.

atoms		AM1	HF/STO-3G	HF/6-31G	B3LYP/6-31G*
1	N	-1.366955	-1.565772	-0.928144	-0.463849
2	C	0.414306	0.338409	0.359168	0.228822
3	C	0.427113	0.383298	0.360676	0.230521
4	N	-1.217105	-1.565786	-0.928144	-0.463849
5	C	0.344703	0.338414	0.359168	0.228822
6	C	0.34695	0.383302	0.360676	0.230521

Table S2. Calculated partial charge of dimethylamino carbon atoms of five reported haptens.

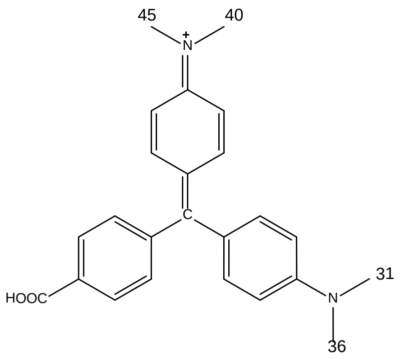

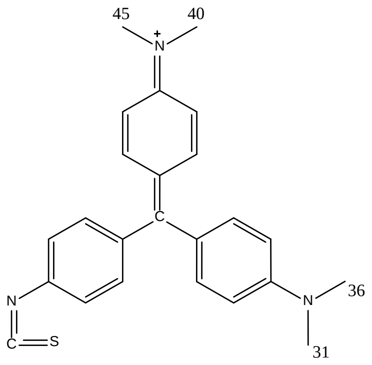

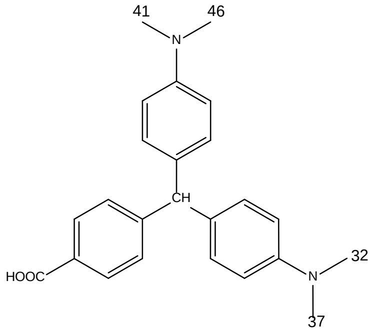

A			B			C		
31	C	0.233730	31	C	0.230090	32	C	0.156683
36	C	0.235630	36	C	0.228719	37	C	0.156024
40	C	0.232989	40	C	0.228749	41	C	0.157618
45	C	0.235172	45	C	0.230224	46	C	0.156384

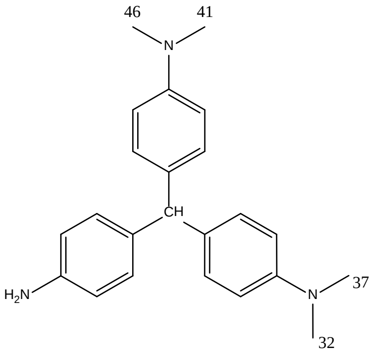

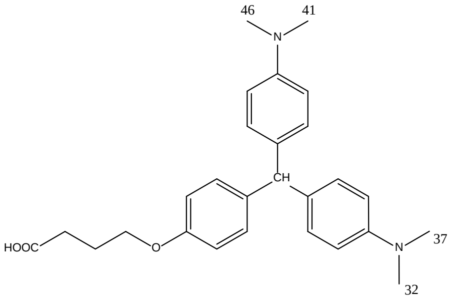

D			E		
32	C	0.151416	32	C	0.154602
37	C	0.150437	37	C	0.154195
41	C	0.150399	41	C	0.154641
46	C	0.151539	46	C	0.155146

Table S3. Calculated partial charge of dimethylamino carbon atoms of TDs analogues from SciFinder, ACD or Zinc database.

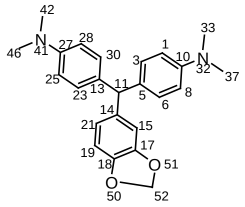 21072011_133601			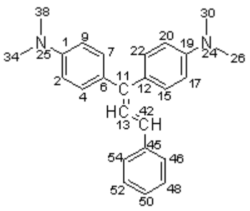 21072011_144018			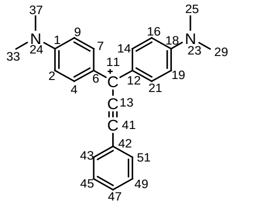 21072011_144235		
33	C	0.152416	26	C	0.156409	25	C	0.228808
37	C	0.153087	30	C	0.156648	29	C	0.227721
42	C	0.152927	34	C	0.155104	33	C	0.227721
46	C	0.152151	38	C	0.154922	37	C	0.228808
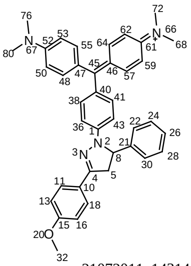 21072011_143144			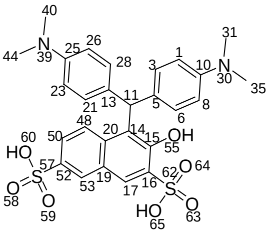 21072011_133159			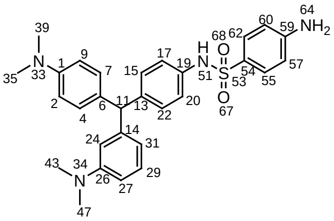 21072011_141628		
68	C	0.211749	31	C	0.157249	35	C	0.153237
72	C	0.211565	35	C	0.158332	39	C	0.152950
76	C	0.210805	40	C	0.156130	43	C	0.157305
80	C	0.211220	44	C	0.155910	47	C	0.150799
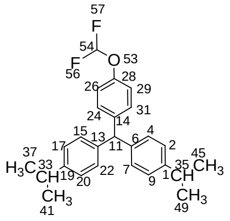 21072011_132958			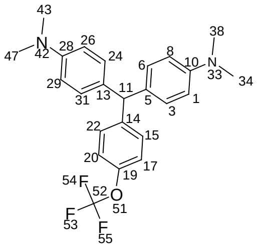 21072011_133035			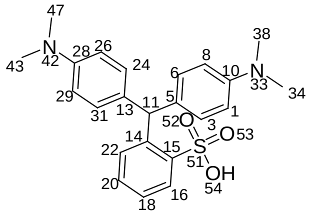 21072011_133231		
37	C	-0.004178	34	C	0.155869	34	C	0.153310
41	C	-0.004651	38	C	0.154652	38	C	0.154470
45	C	-0.004410	43	C	0.155245	43	C	0.153443
49	C	-0.004167	47	C	0.154802	47	C	0.153351
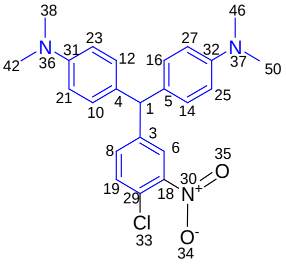 MFCD00100147			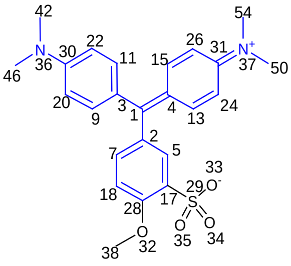 MFCD00144834			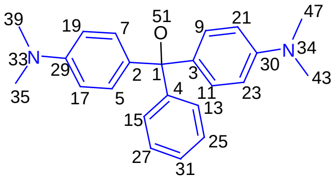 MFCD00151208		

38	C	0.158490	42	C	0.194282	35	C	0.156307
42	C	0.158268	46	C	0.191635	39	C	0.158011
46	C	0.158512	50	C	0.209925	43	C	0.153389
50	C	0.159161	54	C	0.196617	47	C	0.154595

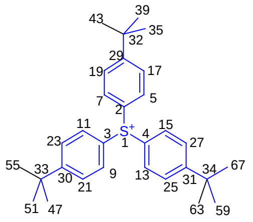
MFCD02683475

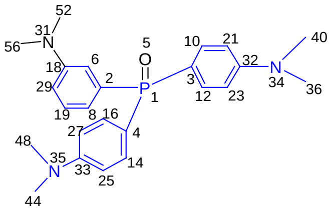
MFCD02177828

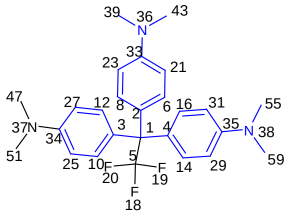
MFCD00813906

35	C	0.007204	36	C	0.163356	39	C	0.156358
39	C	0.020663	40	C	0.160938	43	C	0.155816
43	C	0.024351	44	C	0.161189	47	C	0.156696
47	C	0.007517	48	C	0.163395	51	C	0.157039
51	C	0.020909	52	C	0.166779	55	C	0.156060
55	C	0.024479	56	C	0.153173	59	C	0.156618
59	C	0.021025						
63	C	0.007314						
67	C	0.024232						

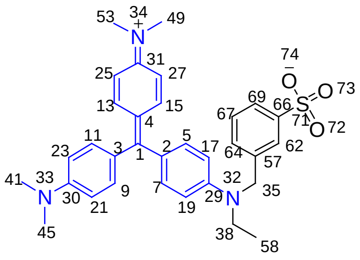
MFCD00166979

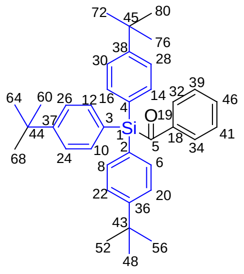
MFCD03931987

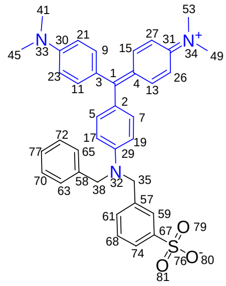
MFCD00068428

41	C	0.188905	48	C	-0.007497	41	C	0.189165
45	C	0.188305	52	C	-0.022254	45	C	0.225108
49	C	0.191707	56	C	-0.009972	49	C	0.191031
53	C	0.244071	60	C	-0.010685	53	C	0.192583
			64	C	-0.023857			
			68	C	-0.011524			
			72	C	-0.023187			
			76	C	-0.010893			
			80	C	-0.007577			

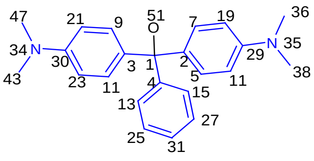
MFCD00012483

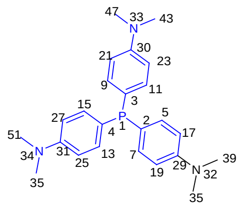
MFCD00014866

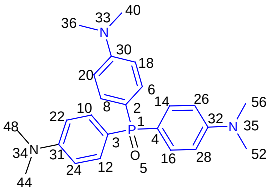
MFCD00025649

35	C	0.153389	35	C	0.156463	36	C	0.160255

39	C	0.154595	39	C	0.155951	40	C	0.163242
43	C	0.158010	43	C	0.156205	44	C	0.162739
47	C	0.156304	47	C	0.155612	48	C	0.159910
						52	C	0.162778
						56	C	0.159986

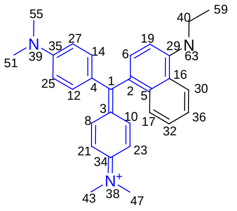
MFCD00011876

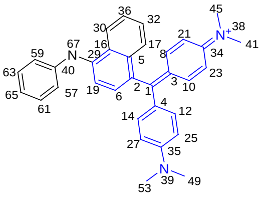
MFCD00011878

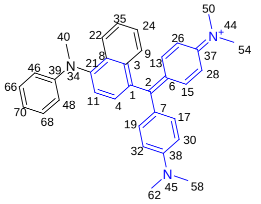
MFCD00036331

43	C	0.216765	41	C	0.217800	50	C	0.217060
47	C	0.217281	45	C	0.218154	54	C	0.216619
51	C	0.214926	49	C	0.215667	58	C	0.218236
55	C	0.215507	53	C	0.216447	62	C	0.218932

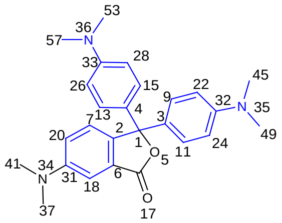
MFCD00070611

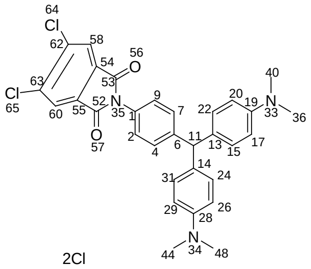
2Cl

37	C	0.170124	36	C	0.154451
41	C	0.160380	40	C	0.153844
45	C	0.154495	44	C	0.154876
49	C	0.156577	48	C	0.153702
53	C	0.158690			
57	C	0.157325			

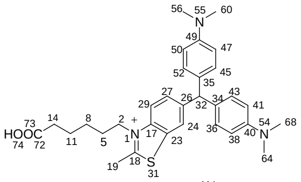
1N

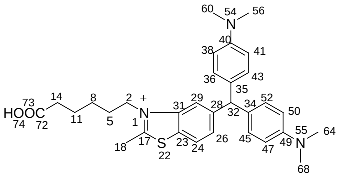
2N

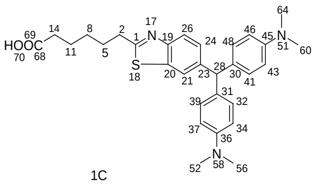
1C

56	C	0.172212	56	C	0.173441	52	C	0.154600
60	C	0.176510	60	C	0.181802	56	C	0.155000
64	C	0.173922	64	C	0.175572	60	C	0.154124
68	C	0.172447	68	C	0.175278	64	C	0.154542

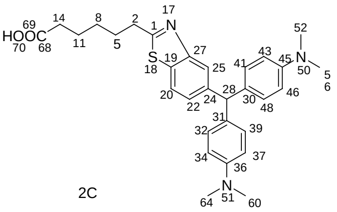
2C

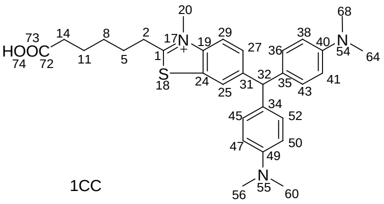
1CC

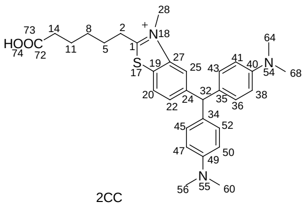
2CC

52	C	0.154503	56	C	0.174610	56	C	0.172658
56	C	0.154926	60	C	0.174031	60	C	0.172186
60	C	0.154619	64	C	0.172120	64	C	0.173224
64	C	0.155231	68	C	0.177219	68	C	0.171255

Table S4. Calculated partial charges of haptens' carbon atoms for the designed haptens.

a) with simple modification

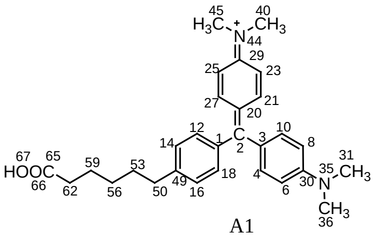
A1

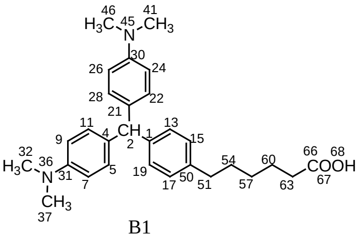
B1

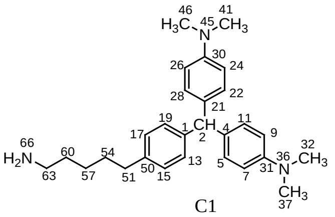
C1

31	C	0.226104	32	C	0.152674	32	C	0.151659
36	C	0.227602	37	C	0.152005	37	C	0.152183
40	C	0.225762	41	C	0.151952	41	C	0.151901
45	C	0.227075	46	C	0.153184	46	C	0.152705

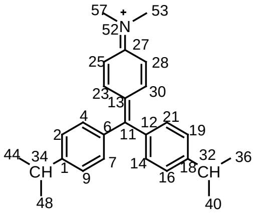
CVC

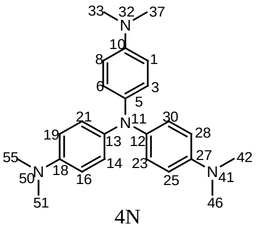
4N

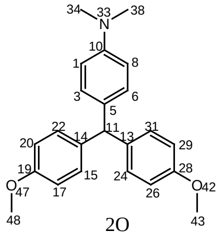
2O

36	C	0.030755	33	C	0.147758	34	C	0.153216
40	C	0.030767	37	C	0.148349	38	C	0.153742
44	C	0.030755	42	C	0.147731	43	C	0.254175
48	C	0.030767	46	C	0.148262	48	C	0.253940
53	C	0.241280	51	C	0.147875			
57	C	0.241280	55	C	0.148460			

b) with electron donating group for oxidized form TDs

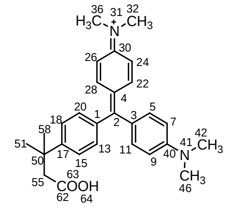
phe0

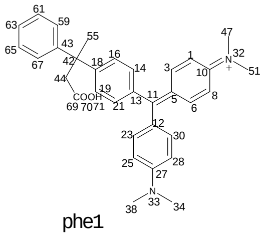
phe1

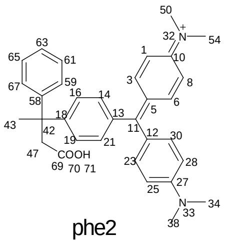
phe2

32	C	0.225636	34	C	0.226994	34	C	0.226079
36	C	0.226451	38	C	0.224763	38	C	0.227357
42	C	0.227441	47	C	0.225300	50	C	0.227773
46	C	0.225166	51	C	0.224286	54	C	0.226470

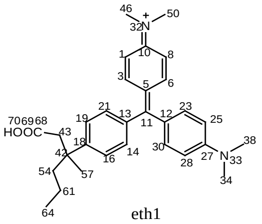
eth1

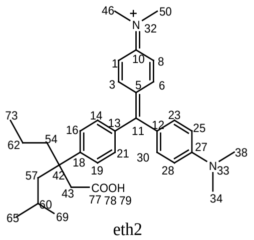
eth2

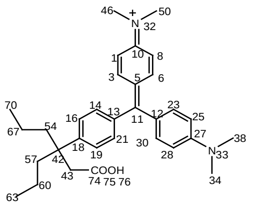
2ETH

34	C	0.225739	34	C	0.225122	34	C	0.226198
38	C	0.229821	38	C	0.226288	38	C	0.224792

46	C	0.225970	46	C	0.227461	46	C	0.226076
50	C	0.225331	50	C	0.225981	50	C	0.224996

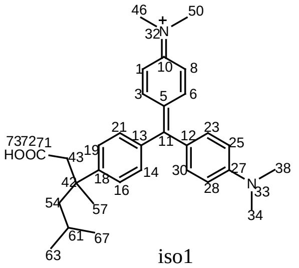

iso1

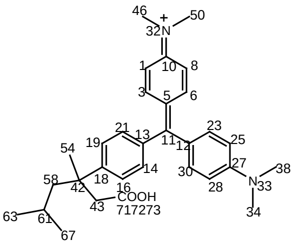

iso2

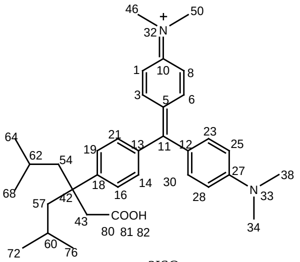

2ISO

34	C	0.225135	34	C	0.226549	34	C	0.281606
38	C	0.228727	38	C	0.225338	38	C	0.279353
46	C	0.225199	46	C	0.227335	46	C	0.279900
50	C	0.224542	50	C	0.226154	50	C	0.278811

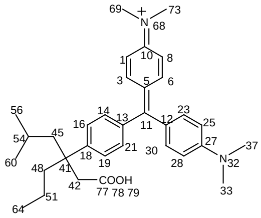

ie1

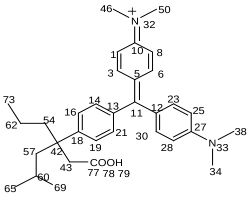

ie2

33	C	0.223948	34	C	0.225402
37	C	0.225779	38	C	0.224145
69	C	-0.013546	46	C	0.225763
73	C	0.004094	50	C	0.224761

c) with electron withdrawing group for reduced form TDs

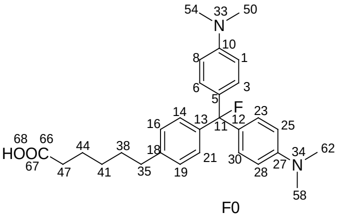

F0

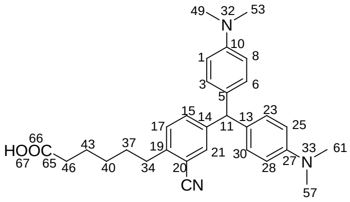

CN

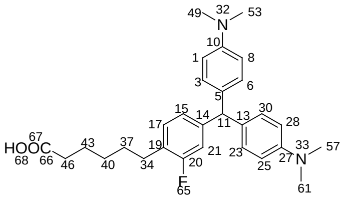

F

50	C	0.156452	49	C	0.156169	49	C	0.154421
54	C	0.155477	53	C	0.156026	53	C	0.153093
58	C	0.155636	57	C	0.156622	57	C	0.154284
62	C	0.156204	61	C	0.156383	61	C	0.153328

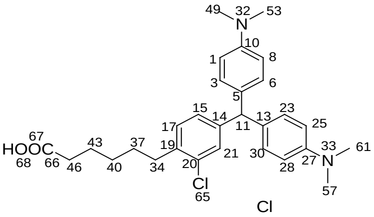

Cl

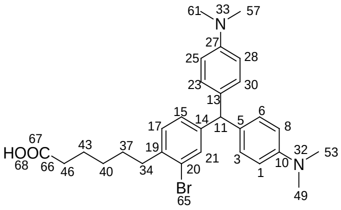

Br

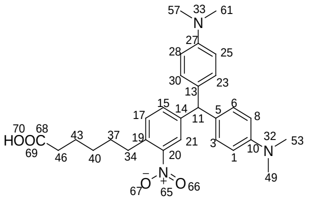

NO2

49	C	0.155137	49	C	0.155067	49	C	0.156698
53	C	0.153799	53	C	0.153737	53	C	0.156531
57	C	0.154891	57	C	0.154972	57	C	0.156784
61	C	0.154142	61	C	0.154122	61	C	0.156766

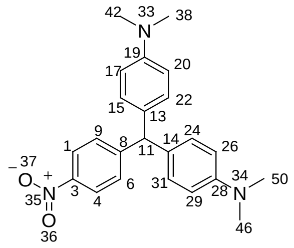

NOO

38	C	0.157667
42	C	0.158686
46	C	0.157551
50	C	0.157857

d) with N⁺ electron withdraw groups for oxidized form TDs

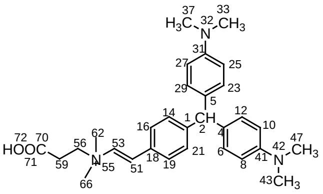

co0

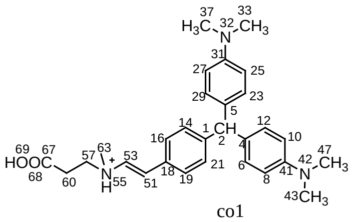

co1

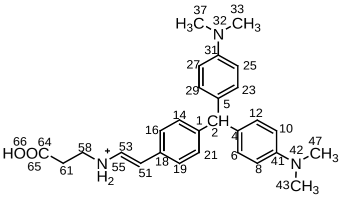

co2

33	C	0.171242	33	C	0.168745	33	C	0.169345
37	C	0.166441	37	C	0.167911	37	C	0.167934
43	C	0.167129	43	C	0.171481	43	C	0.172271
47	C	0.168775	47	C	0.166406	47	C	0.167207

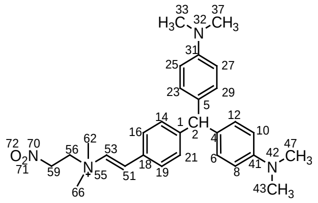

no0

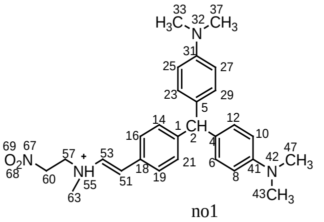

no1

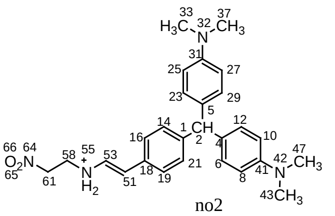

no2

33	C	0.166087	33	C	0.169794	33	C	0.170340
37	C	0.171436	37	C	0.169081	37	C	0.169414
43	C	0.167404	43	C	0.172819	43	C	0.168430
47	C	0.168415	47	C	0.167485	47	C	0.173488

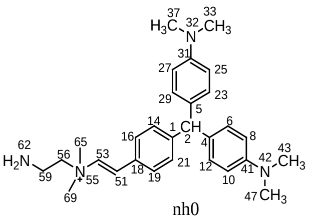

nh0

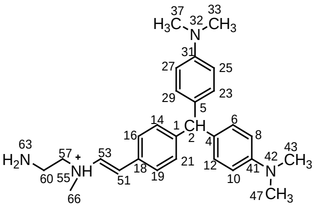

nh1

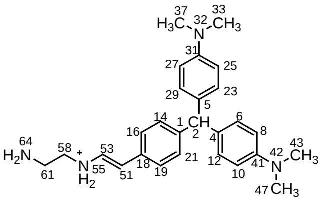

nh2

33	C	0.170494	33	C	0.168521	33	C	0.169093
37	C	0.165265	37	C	0.167558	37	C	0.167576
43	C	0.166478	43	C	0.171216	43	C	0.171874
47	C	0.167635	47	C	0.166289	47	C	0.166925

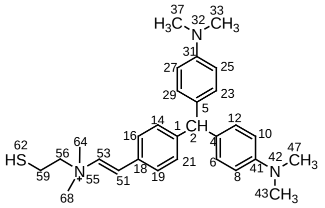

sh0

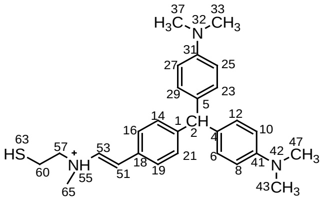

sh1

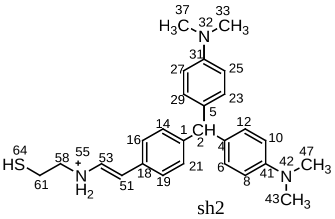

sh2

33	C	0.171480	33	C	0.169049	33	C	0.172449
37	C	0.166369	37	C	0.168174	37	C	0.167207
43	C	0.167937	43	C	0.171839	43	C	0.168682
47	C	0.168928	47	C	0.166811	47	C	0.169421

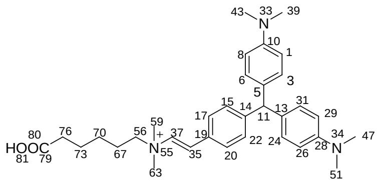

N2

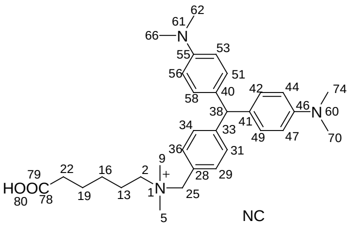

NC

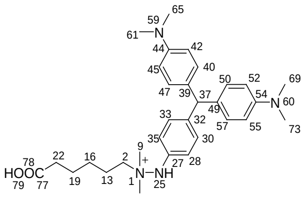

NNH

39	C	0.170105	62	C	0.170394	61	C	0.168287
43	C	0.164528	66	C	0.166587	65	C	0.171517
47	C	0.167038	70	C	0.168363	69	C	0.170615
51	C	0.166533	74	C	0.171802	73	C	0.166524

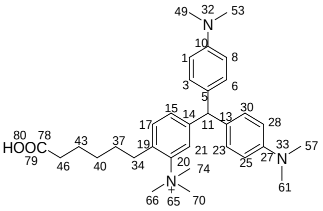

N

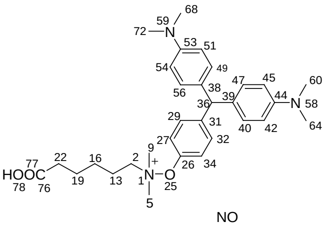

NO

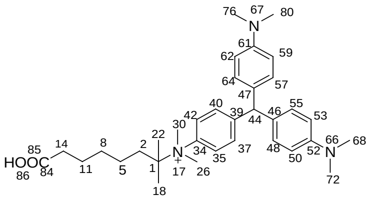

CN1

49	C	0.172261	60	C	0.199318	68	C	0.170571
53	C	0.177335	64	C	0.200997	72	C	0.169572
57	C	0.174581	68	C	0.194599	76	C	0.168176
61	C	0.174055	72	C	0.193333	80	C	0.173469

59	C	0.171082	59	C	0.170810	62	C	0.172528
63	C	0.169480	63	C	0.170822	66	C	0.167533
67	C	0.172691	67	C	0.168898	70	C	0.168038
71	C	0.168395	71	C	0.175269	74	C	0.169410

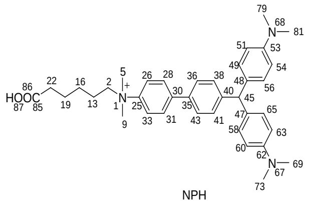 NPH						
69	C	0.165927	65	C	0.173222	0.175581
73	C	0.161498	69	C	0.168625	0.170265
77	C	0.162523	73	C	0.169289	0.171459
81	C	0.164074	77	C	0.170825	0.172705

Table S5. Structure-cross reactivity relationship of the hapten with TDs.

Compounds	Average charge of C atoms	Absolute charge difference*	Percentage difference %**	Cross-reactivity (rabbit)	Cross-reactivity (mice)
MG	0.229671	0.057	33.14	24.50	20.56
LMG	0.152528	0.020	11.58	51.22	37.79
CV	0.216933	0.044	25.75	29.24	23.49
LCV	0.150946	0.021	12.50	68.73	55.64
-N+	0.172503	0	0	100%	100%

* Charge difference of the indicative C atoms with hapten (–N+).

** The charge difference in percentage by dividing the charge of hapten (0.172503).

Table S6. Working dilution of coating antigen and secondary antibody by checkerboard test (n=3).

	Dilution of IgG secondary antibody	Concentration of the coating antigen(μg•mL^{-1})				
		0.1	0.2	0.5	1	2
anti rabbit IgG	5000	0.813	0.813	0.814	0.991	0.757
	10000	0.506	0.542	0.566	0.598	0.476
anti mouse IgG	5000	0.493	0.535	0.594	0.691	0.657
	10000	0.706	0.782	0.866	0.998	0.776
